# Population estimation beyond counts—Inferring demographic characteristics

**DOI:** 10.1371/journal.pone.0266484

**Published:** 2022-04-05

**Authors:** Noée Szarka, Filip Biljecki

**Affiliations:** 1 School of GeoSciences, University of Edinburgh, Edinburgh, United Kingdom; 2 Department of Architecture, National University of Singapore, Singapore, Singapore; 3 Department of Real Estate, National University of Singapore, Singapore, Singapore; University of Wisconsin Madison, UNITED STATES

## Abstract

Mapping population distribution at a fine spatial scale is essential for urban studies and planning. Numerous studies, mainly supported by geospatial and statistical methods, have focused primarily on predicting population counts. However, estimating their socio-economic characteristics beyond population counts, such as average age, income, and gender ratio, remains unattended. We enhance traditional population estimation by predicting not only the number of residents in an area, but also their demographic characteristics: average age and the proportion of seniors. By implementing and comparing different machine learning techniques (Random Forest, Support Vector Machines, and Linear Regression) in administrative areas in Singapore, we investigate the use of point of interest (POI) and real estate data for this purpose. The developed regression model predicts the average age of residents in a neighbourhood with a mean error of about 1.5 years (the range of average resident age across Singaporean districts spans approx. 14 years). The results reveal that age patterns of residents can be predicted using real estate information rather than with amenities, which is in contrast to estimating population counts. Another contribution of our work in population estimation is the use of previously unexploited POI and real estate datasets for it, such as property transactions, year of construction, and flat types (number of rooms). Advancing the domain of population estimation, this study reveals the prospects of a small set of detailed and strong predictors that might have the potential of estimating other demographic characteristics such as income.

## Introduction

With more than half of the world’s population living in urban areas, and with this trend continuing positive trajectory, urban management, planning and analysis are increasingly important to better understand, manipulate and improve urban systems [1–3]. For effective planning and appropriate measures, data on demographic distributions plays an important role [2, 4]. These spatial patterns are essential to gain knowledge about socio-economic and environmental phenomena, which supports both public and private sectors in planning and decision making [5, 6]. Demographic counts are usually provided by population censuses, which enable identifying patterns of human distribution at administrative units [7]. However, these censuses can be expensive, they are usually conducted at low temporal resolution, and they are fixed at zones at a certain spatial scale, which can lead to biases as part of the modifiable area unit problem [8–10]. Hence, it is crucial to develop different approaches and methods with the help of GIS and statistics to overcome some of these issues, primarily with the goal of providing reliable demographic data at a fine spatial scale. Such datasets may be found useful for a variety of applications, e.g. energy demand estimations [11], health studies [12], planning amenities [13], and waste management [14].

There is a long history of population estimation in GIS. Areal interpolation is a well-tried way to disaggregate population numbers from larger to smaller areas or administrative levels, for example, by simple area weighting or dasymetric mapping [6, 8, 15–18]. In contrast, another approach is to establish statistical relationships between population and certain spatial information in a number of zones, and use regression to estimate the population in other areas at the same administrative or spatial scale level [19, 20].

Both approaches have been applied in studies for the estimation of population in small areas, being driven by one or more multiple predictors that hint at the size of the population [5, 21]. These predictors come in different forms and shapes and from different sources [22]. For example, land use classes and night time lights, derived from remote sensing techniques, are a common set of information that are used in population estimations [1, 4, 23–25]. Further examples are many: household counts [4, 6], telecommunication data [10, 26, 27], tax parcel information [28], and social media [29, 30]. The large number of disparate information and wide range of data sources used in the analyses are united in predicting the number of people living in an area, but they do not do much beyond that despite the diversity of input data.

As previous work focuses almost entirely on predicting population numbers only, there is a gap in research in accompanying population count estimation by also inferring demographic or socio-economic patterns of people behind those counts, such as age, gender, and income. This is important because, as our study will affirm, subdivisions of large areas often have heterogeneous population characteristics, besides having diverging population counts. The same set of applications that use spatial population data, could appreciate the availability of an expanded set of information such as demographic characteristics [7, 31]. For example, demographic characteristics and not just population counts are important in epidemiology [32, 33] and in estimating energy consumption behaviour [34, 35].

In this paper, we investigate how can population estimation techniques be expanded to include inferring demographic attributes as well. In our study, we have focused on predicting the age of residents, an especially important demographic characteristics nowadays. For example, the age of residents in an area may be relevant for a number of use cases such as urban planning and business intelligence. Further, rapidly ageing societies pose many future challenges, which are eminent for well-developed geographies such as our study area—Singapore [36–38]. Hence, appropriate measures regarding eldercare, retirement, and transport (among many others) need to be addressed, which are unexceptionally bound to geographical patterns [39–41], and can be supported by spatial data detailing demographic distributions.

To the extent of our knowledge, the work of [42] is the only study which has aimed to predict demographic structures so far, by estimating the numbers of children under 5 years across Nigeria with the help of land cover, night time lights, vegetation index and travel time to major settlements, for the purpose of developing vaccination strategies. Our work differs from theirs by estimating the average age of residents, by focusing on senior population, and by using a different set of data—we are focusing on real estate and point of interest (POI) data, rather than data derived from remote sensing, presenting a contribution in this domain.

During our research, we have encountered further research opportunities in the traditional population estimation, which we attempt to bridge in this paper. These research gaps and aims are elaborated in the continuation of the paper, with the two most important as follows.

First, we notice that some POI (i.e. amenities; in our paper we use the two terms interchangeably) and real estate data we have at our disposal not only have not been used to predict age patterns, but they have also not been used in population count estimation. For that reason, we include also traditional population count estimation, as an intermediate step towards our enhanced demographic-aware population estimation. The selection of these datasets follows our hypothesis that amenities and real estate in neighbourhoods have been shaped by the demographics of its residents, a reasoning that has been inspired by recent work using such data for population estimation [18, 43, 44]. Hence our work also contributes to the body of knowledge by uncovering the value of different amenity and real estate data in population count, besides inferring demographic characteristics.

Second, as our work largely relies on machine learning (ML), we pay special attention in understanding how do different ML techniques differ in their accuracy of predicting demographic patterns. In our work, instead of merely identifying the most effective technique in the exploratory phase, we conduct the analysis using multiple approaches, which is a contribution considering that comparative analyses are seldom in this domain and given that we provide potentially valuable insights to other researchers in population counts in suggesting reliable techniques for population estimation.

Finally, while we focus on one demographic attribute, we believe that our work could be expanded to cover other key ones such as income, gender ratio, and ethnicity, as well.

## Background and related work

### Point features, and amenities/POI and real estate data

Point-based features (i.e. when the location of a real-world feature is represented by a point) have been frequently included in disaggregation research, due to its simple data structure and wide availability [45]. In our research, we focus on two instances of point-based features: points of interest and real estate data. The latter domain of data is of wide variety coming in different geometric forms (e.g. building footprints as polygons), but as it will explained later, in our research, we focus solely on point-based real estate data.

POIs such as schools, banks, bus/metro stations, clinics, parking lots, restaurants and museums have proven to have a considerable relevance with population patterns and often correlate with density, and hence have been used in population studies [24, 46, 47]. Another advantage of these features is that they can often be easily obtained from datasets openly released by national mapping agencies or from Volunteered Geographic Information (VGI), i.e. OpenStreetMap [1, 6, 46, 48]. Our work extends related instances with the hypothesis that the density of particular amenities that caters to a specific demographic group may be useful as a predictor of age, i.e. amenities in a neighbourhood will reflect its residents’ demographics. For example, we expect that neighbourhoods with a higher number of schools, will have a population younger than the national average. Furthermore, we investigate the inclusion of other amenities that, to the extent of our knowledge, have not been used in related work.

Geospatial real estate and housing stock data has been included in the analysis, since it has repeatedly proven to be significant in previous population estimation approaches [6, 15], and has been extensively linked to demography in other studies [49–51]. Examples of housing predictors that have been used are the number of buildings, their footprint area, floor area, and volume [20, 47, 52, 53]. Nowadays, real estate datasets are available from commercial websites or the government, and may support population estimation methods significantly [15, 28, 54]. However, there are other types of data related to real estate that have not been used in such studies, such as property transactions and age of buildings (i.e. year of construction). Thus, we believe that it is important to investigate their role in population estimation, which we focus on in our study.

### Machine learning in population estimation

The rise of ML algorithms has also made its mark into GIS applications. While linear regression has been applied in geographical analysis for decades, more sophisticated methods have become popular in recent years. In particular, Random Forest (RF), a supervised ML algorithm based on decision trees, has evolved into the researchers’ favourite method in estimating population counts [1, 24, 48]. Alternatively, Support Vector Machines (SVM), which aim to find an ideal hyperplane in an multi-dimensional space, have been widely employed especially in remote sensing, and furthermore in hyper-complex applications such as facial recognition [55–57]. With the exception of the work of [4], SVM however still leads a shadow existence in population predictions, despite its efficient implementation in other studies.

## Data and methods

### Overview of the approach

In this paper, we focus on estimating the age aspect as one of the most important demographic characteristic. More specifically, we predict the average age of residents in a district and the percentage of seniors (65 years and above). As expected, these two attributes are highly correlated (in our case, based on the data and study area that will be introduced in a bit, the correlation coefficient is 0.97), but we have decided to include both since each might be found useful and so that we provide more than one age/demographic characteristic.

The selection of the ancillary data is influenced by both the availability of the data in our study area and based on the literature review, giving priority to latent data that has not been used before.

In the estimations, our method mirrors a typical regression development: we use data of a limited set of administrative areas as training dataset, and test the performance of the developed regression model on a set of different areas at the same administrative level. We put much focus on providing a comparative overview of multiple machine learning approaches. Thus, we implement three methods: random forest, support vector machines, and linear regression.

Because the secondary contribution of our work is to investigate the effects of latent data on amenities and real estate for traditional population estimation, we also infer population counts, before predicting the average age and proportion of seniors in an administrative area. The combination of population counts and socio-economic numbers may be useful to combine, e.g. to calculate the total number of seniors.

**Fig 1:**
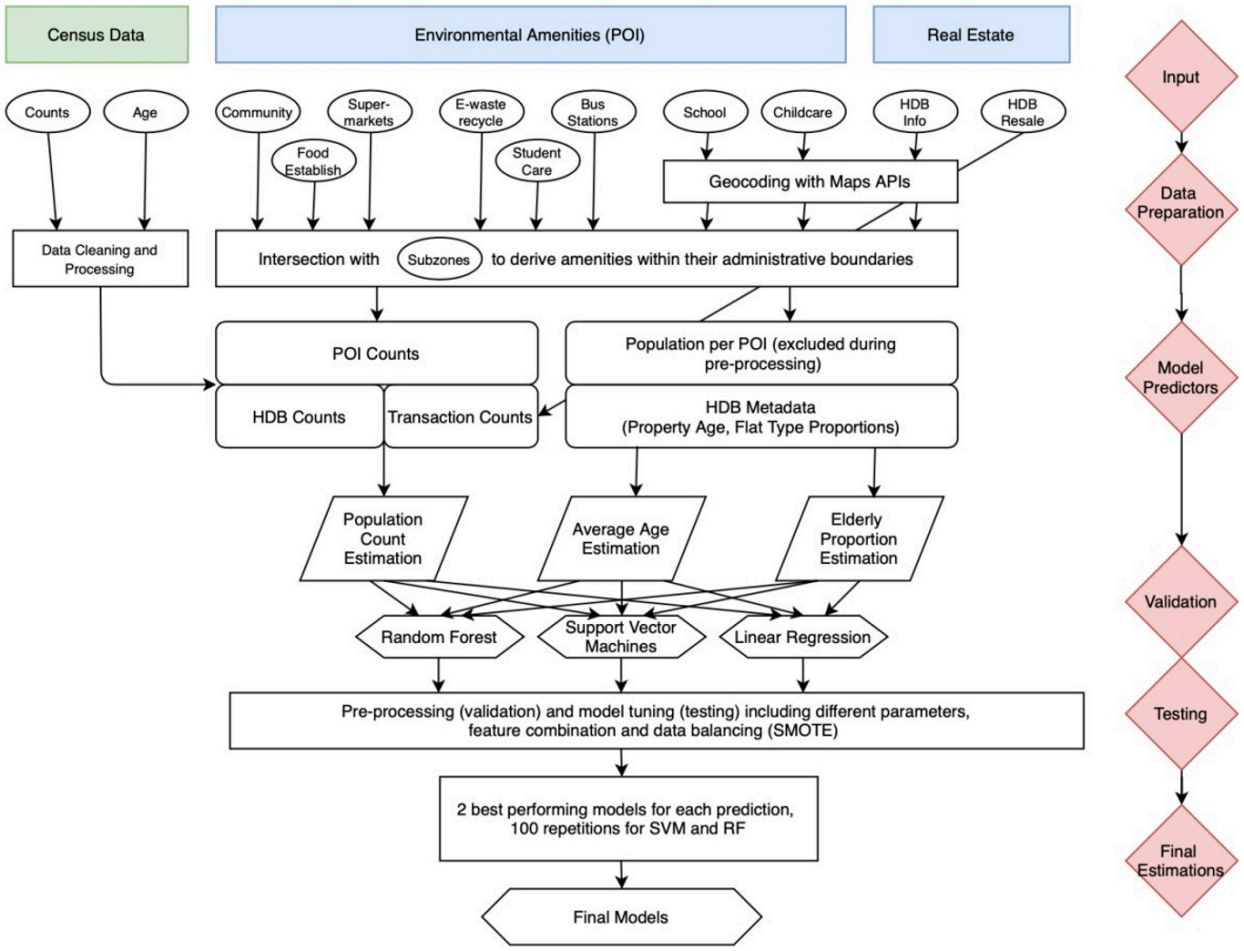
Detailed flowchart of the method and the employed datasets.

The overview of the work is illustrated in Fig 1.

### Study area

The study area enfolds the so-called HDB (Housing & Development Board—Singapore’s public housing authority) towns and estates in Singapore, a city-state in Southeast Asia. Approximately 80% of residents in Singapore live in flats developed and managed by HDB, of which about 90% own their property [58]. There is a range of real estate data available for these properties and towns, facilitating our research.

These highly urban regions are characterised by lowland covered by superstructures and high-rise buildings, but also by many green areas such as parks, natural reserves and water catchment areas [58, 59], for which various data is available as well.

**Fig 2:**
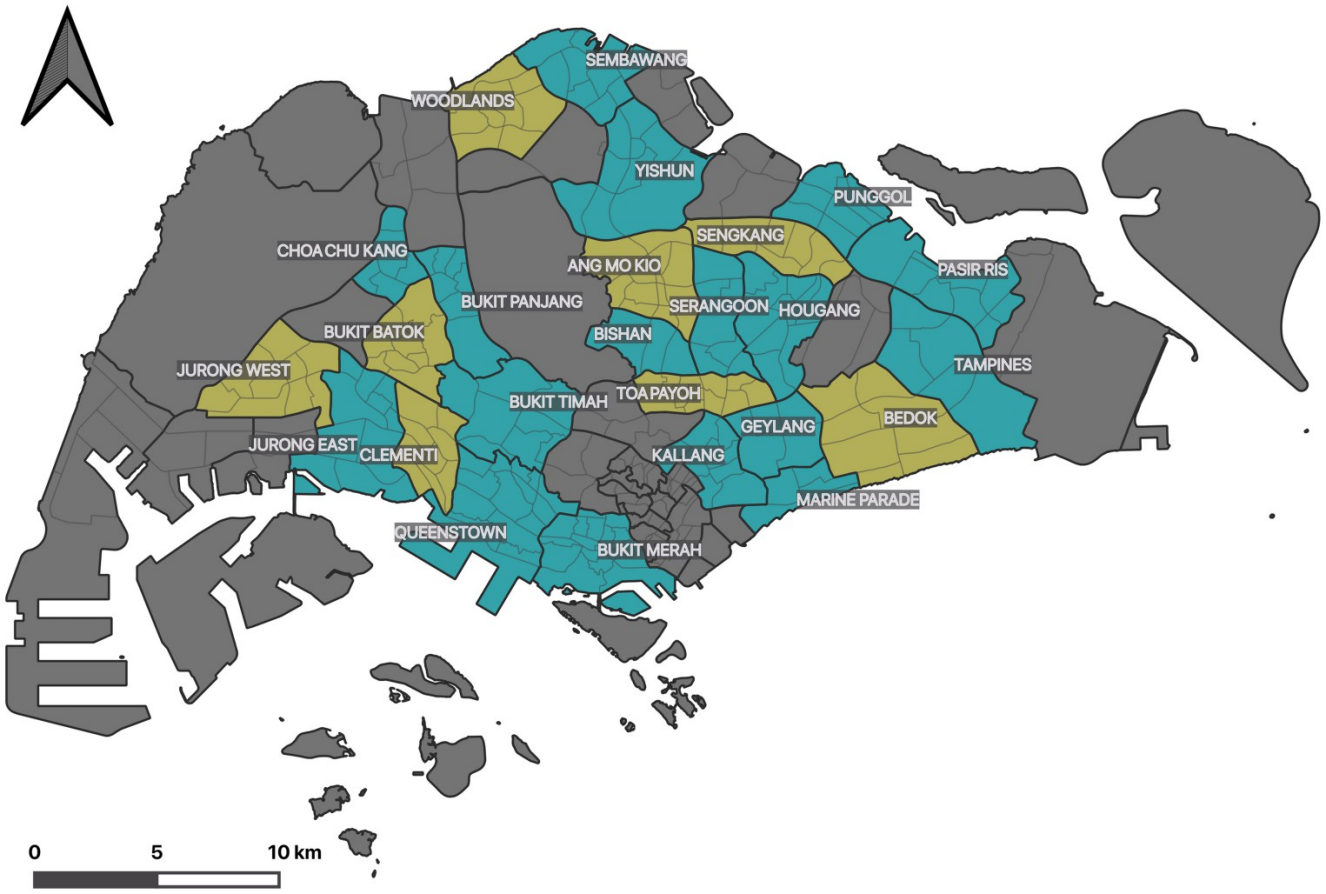
Planning areas (thick lines) including their subzones (thin lines) in Singapore. The yellow areas are part of the training group, while the turquoise zones are the test areas for estimations. The grey parts of the country are out of scope of our work because they are not residential or not dominated by HDB. Source of the administrative dataset: Urban Redevelopment Authority / data.gov.sg (2014).

Administratively, Singapore is divided into 55 planning areas, and each is further subdivided in multiple subzones, which is the smallest administrative entity in the city-state and it is in intended for statistical purposes. In total, there are 323 subzones, and in our research, we zero in on this administrative level. Because we focus on planning areas that are largely inhabited by residents living in public housing buildings (also known as *HDB blocks*), in total 215 subzones are part of this work (Fig 2), and we split them for training and testing (75 and 140, respectively). The split has been carried out randomly.

### Data acquisition and preparation, and tools

In our research, we use several datasets on real estate and amenities, which we use as predictors after processing and associating them with subzones (Table 1). The datasets are sourced from open data released by the Singapore Government through the portal data.gov.sg. Some of the data was not available in a geospatial format (e.g. the dataset on the housing stock contains the location each building as address, but not as its spatial coordinates). These have been geocoded using the Google Maps Platform.

**Table 1:** An overview of the predictors. For each subzone, the density of each amenity has been computed.

Predictor	Source
*POI*	
Food establishments	National Environment Agency
Student care services	Ministry of Social and Family Development
Bus stops	Land Transport Authority
Supermarkets	National Environment Agency
Residents committees	People’s Association
E-waste recycling locations	National Environment Agency
Eldercare services	Ministry of Social and Family Development
Clinics	Ministry of Health
Schools	Ministry of Education
Childcare facilities	Early Childhood Development Agency
*Real estate / housing*	
Number of buildings	Housing and Development Board
No. of property transactions in the last 3 years	Housing and Development Board
Age of buildings (mean, median, mode)	Housing and Development Board
Proportion of 1-room flats	Housing and Development Board
Proportion of 2-room flats	Housing and Development Board
Proportion of 3-room flats	Housing and Development Board
Proportion of 4-room flats	Housing and Development Board
Proportion of executive flats	Housing and Development Board

While the POI data is self-explanatory, real estate data might require some elaboration. For each building, the government provides data on the number of apartments by flat type (e.g. 4-room apartment) and the year of its construction (from which we calculated its age). These information were aggregated to the subzone level to provide their averages (e.g. mean age of buildings per subzone, and proportion of each flat type). Furthermore, resale transactions for HDB blocks are available as open data. In this study, for each area, the number of transactions in the past 3 years has been calculated.

**Fig 3:**
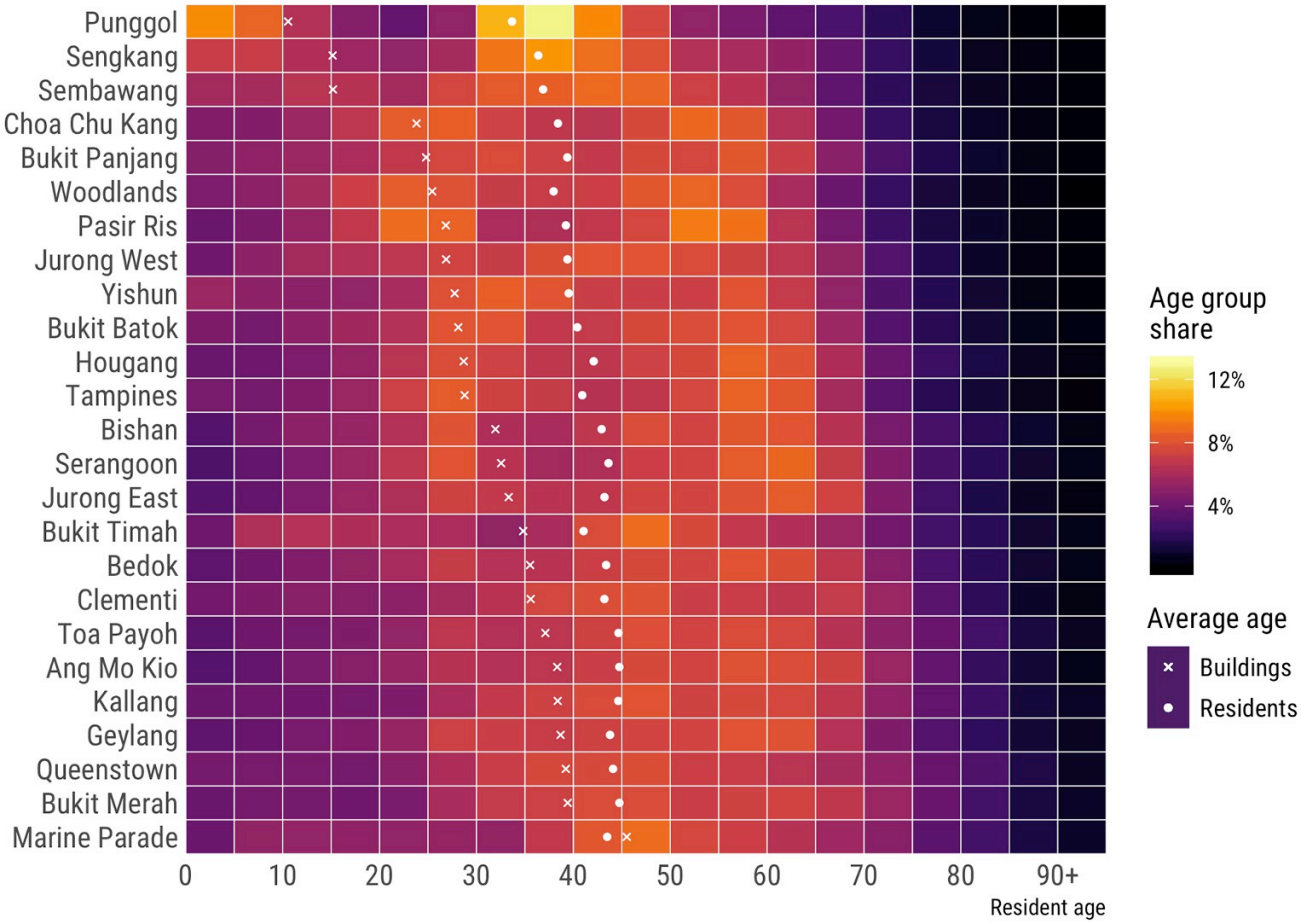
Visualisation of some of the datasets that we have used in our work. Proportion of age groups by administrative area (from which we calculate the proportion of seniors and the average age—plotted as well) together with the average age of buildings. The plot hints at disparate demographics of neighbourhoods and at an association between the age of buildings and age of residents, which we attempt to take advantage of in our estimations. Source of the datasets: Singapore Department of Statistics and Housing and Development Board (data.gov.sg).

The census data has been obtained from an authoritative open dataset [60], from which population counts and age indicators as the dependent variables have been computed. Originally, the dataset contains a fine distribution of population per area by age group (each 5 years of age), as illustrated in Fig 3.

**Fig 4:**
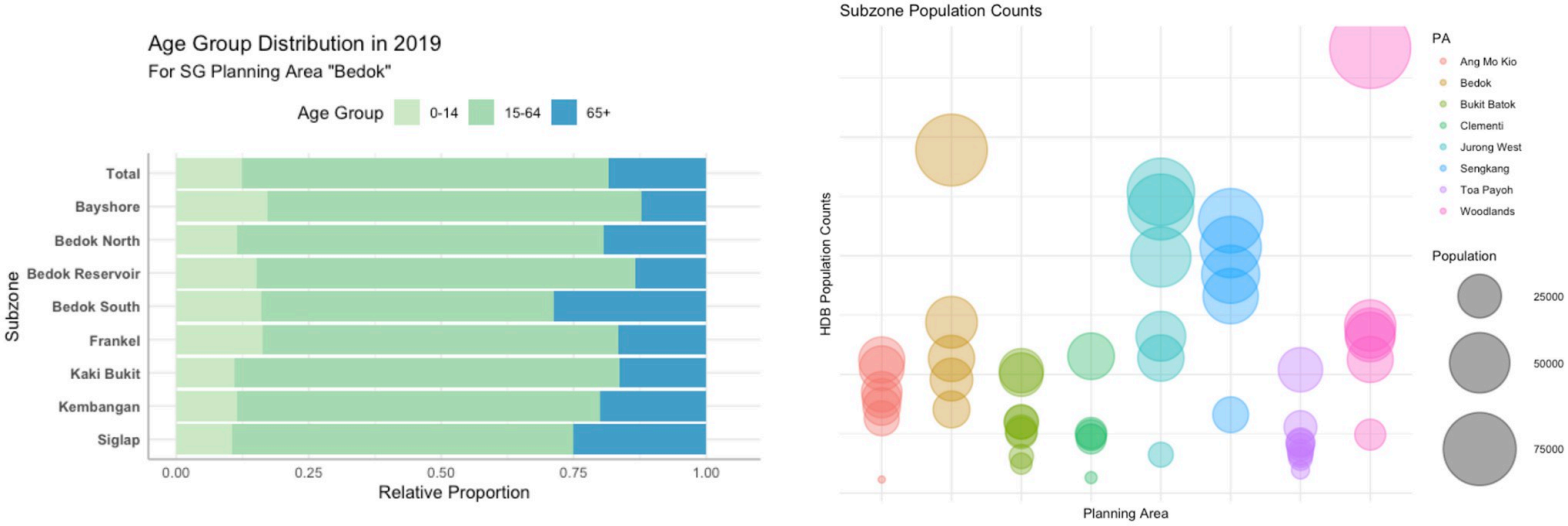
Extracts from the datasets that we have used in our work. (a) aggregated age group distribution for subzones in one of the planning areas in our focus (in our work, we estimate the proportion of the senior group depicted in blue); (b) population counts of subzones are disparate, presenting a suitably diverse dataset for estimations. Source of the datasets: Singapore Department of Statistics and Housing and Development Board (data.gov.sg).

This raw dataset has been transformed into three age groups (see Fig 4) for the purpose of this study as proposed by [61], one of which is elderly (65 years and older), which we select as our focus owing to the increasingly relevant topic of ageing population. The average age has been computed from age groups using the interpolation method of [62]. Both Figs 3 and 4 also suggest the disparate age patterns between areas, affirming the importance of estimating demographic characteristics beyond population counts.

Alternatively, we could have used VGI as the sole source of POI data or to supplement the datasets listed above with additional amenities or their attributes. However, while the completeness of OpenStreetMap data is high in our study area, the semantic content still lacks [63], and we believe that we have a sufficient number of POI categories, so we opted to use only government data. However, in geographies lacking authoritative open data, VGI could be an appropriate source of the same or similar set of datasets.

We implement the work using R. Considering that the tools used are free and open-source, and that datasets we used are available as open data also in many other jurisdictions, this method should be reproducible in other geographical areas.

### Regression models

The regression models have been developed using caret (Classification and Regression Training) in R, which offers a complete framework for data preparation, pre-processing, tuning parameters, training methods and performance analysis [9, 64]. Furthermore, it allows the implementation of different ML algorithms for the same dataset with the same pre-processing parameters, which facilitates model building and comparison [64]. In our work, we use the three previously mentioned techniques, which we briefly explain in the continuation.

Random Forest (RF) is an ensemble supervised machine learning algorithm that makes use of random decision trees [65]. It can be applied to classification and regression problems, in which the latter was relevant for this work. RF for regression is based on *growing trees*: each node in a random forest is split using the best among a subset of predictors randomly chosen at that particular node [66]. Due to the relatively small dataset in our study, the number of trees has been held constant at 1000.

Support Vector Machines (SVM) are a set of optimisation algorithms that construct an ideal hyperplane within an N-dimensional space, in which N is the number of input variables [67]. The support vectors are the closest points of each variable to the hyperplane, and influence its position in space [68]. Similar to RF, the model is suitable to address classification and regression predictions. The key SVM hyperparameters are kernel type and cost (complexity control) [64]. While the cost parameter has not been manipulated because of the low number of variables, a linear kernel has been chosen due to the nature and low complexity of the data [57, 68].

Linear regression is a classic and widely-known method, and compared to RF and SVM, it is relatively simple, resulting in linear models (LM). Instead of working with trees (RF) or hyperplanes and support vectors (SVM), it simply assumes linearity between the independent and dependent variables [69]. The predicted variable is estimated by a weighted linear combination of the covariates (predictors) [70].

The performance of these three approaches in our study will be discussed in the next session.

During the model development stage, feature engineering has been performed to test their effects on model and estimation accuracy [71]. Synthetic samples have been tested for population counts, due to the high variation of residents in subzones (see the right plot in Fig 4). Once the best model tuning parameters have been identified, 100 repetitions have been performed (RF and SVM, not required for LM) for the final estimations of population counts, average age, and the elderly (65 years and above), using the two best models for each prediction. Also, the most highly correlating pairs of variables have been combined into additional new predictors (feature combination; FC) [71].

Training the regression model and interpreting the performance in the pre-testing stage in different scenarios of predictors revealed the overall importance of POIs and real estate data. In the case of all techniques, when it comes to estimating population counts, the R-squared was 0.98. Using POIs only and real estate data only, the values were 0.85 and 0.98, respectively, suggesting the marginal contribution of POIs when combined with real estate data (case of RF; for SVM and LM the results are comparable so they are not all given here, nor in the next paragraph).

In the case of the average age, the R-squared when using real estate data alone was 0.80, while POIs only resulted in 0.45. Combining both sets of predictors did not improve this metric. It is evident that our hypothesis on POIs has been disproved as amenities turn out not to be a relevant predictor of age (at least in our case). Hence, we have decided to exclude POIs from the age estimations. Further, the data on eldercare services has been excluded from the population count estimations because their number was too low to provide insights.

## Results

**Fig 5:**
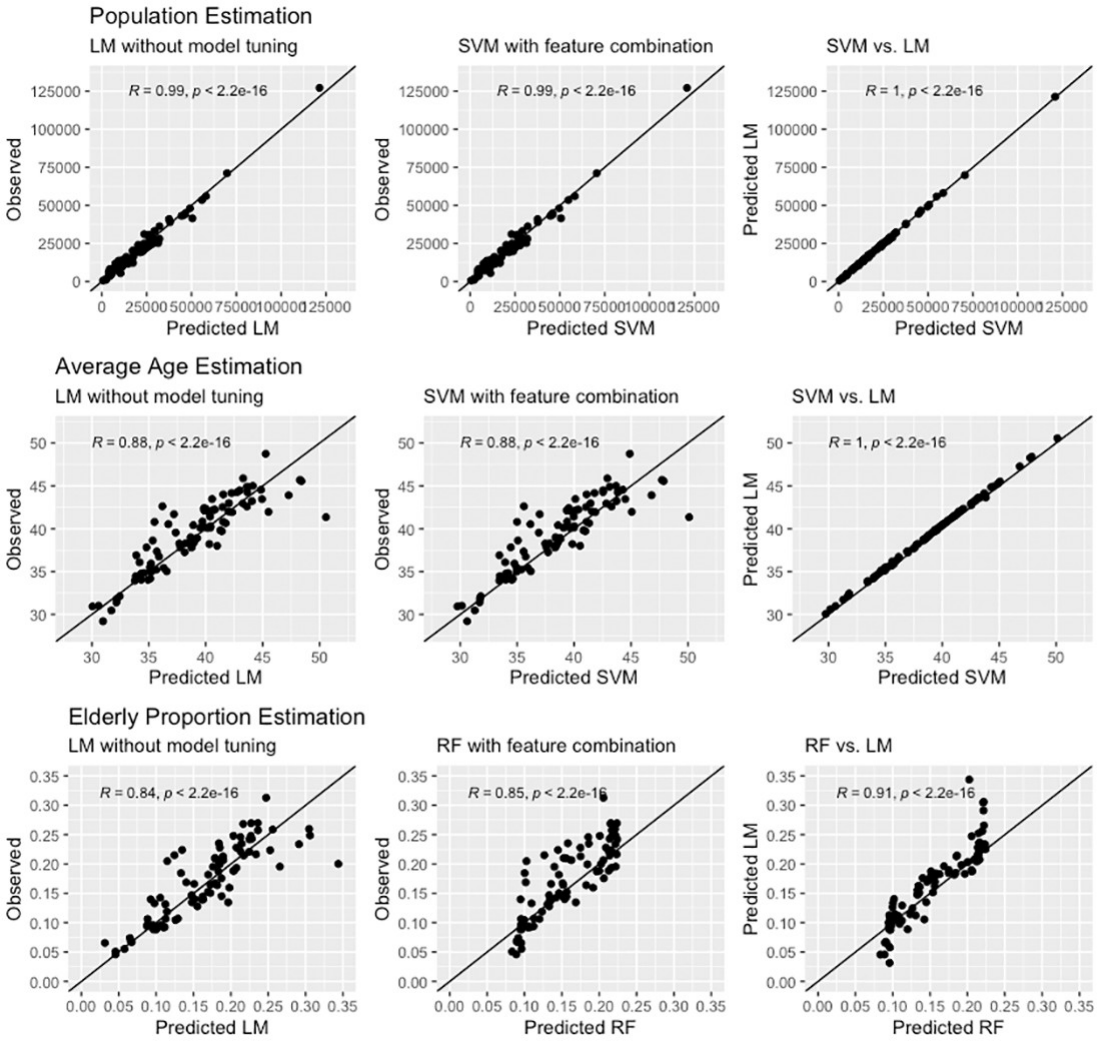
Observed vs predicted and predicted vs predicted (models) scatterplots for population count, average age, and elderly proportion. LM and SVM tend to produce very similar predictions (population counts and average age), while RF and LM reveal differences in particular for lower and higher values (elderly proportion).

The performance of the trained models has been evaluated on the test subzones (denoted in turquoise in Fig 2) and it is given in Table 2 for overview. The models were assessed by R-squared (the explained proportion of the target variable by the predictors), the Mean Absolute Error (MAE), and the Mean Absolute Percentage Error (MAPE) [72, 73]. The estimations have also been interpreted with predicted vs. observed scatterplots (Fig 5) [64, 74, 75]. In general, the results indicate that it is possible to estimate population characteristics in a similar fashion as population estimation. The best performing model is able to predict the average age of residents in a subzone at an MAE of 1.5 years. To put that number in context, the range of average age in the subzones is from 31 to 45, hence it may be considered as an accurate outcome.

**Table 2:** Overview of the performance of the different combinations of the developed regression models to estimate population counts and age.

		RF			SVM			LM		
		R^2^	MAE	MAPE	R^2^	MAE	MAPE	R^2^	MAE	MAPE
Population counts	No model tuning With FC	0.910	3415	0.390	0.973	2441	0.167	0.980	2297	0.173
		0.907	3087	0.332	0.974	2443	0.164	0.974	2469	0.179
Average age	No model tuning With FC	0.745	1.681	0.041	0.767	1.637	0.036	0.768	1.513	0.038
		0.745	1.682	0.044	0.767	1.570	0.040	0.497	1.811	0.042
Senior proportion	No model tuning With FC	0.724	0.029	0.211	0.684	0.063	0.837	0.713	0.026	0.167
		0.728	0.028	0.206	0.695	0.063	0.837	0.446	0.032	0.160

SVM and LM produce nearly equal measures for population numbers, while LM yields the best estimations for average age, a position challenged by RF when it comes to inferring the proportion of senior residents. While the two demographic indicators are closely related and highly correlated, it is interesting to observe differing performance in predicting them.

### Assessment

#### Population count

Throughout the testing phase, LM and SVM have outperformed RF in predicting population counts. In terms of model performance, feature engineering did not have a significant effect. For estimation performance (on the test dataset), feature engineering improved the RF and SVM models. Overall, LM has performed best, and did so without any model tuning (R-squared of 97% for estimation performance).

#### Average age and the share of the elderly

POIs were excluded from age predictions due to their low importance (a key result of the research). Furthermore, these models would have relied on the population estimations from the previous models to calculate the amenity proportion, which would have biased the estimations as a source of error. Hence, only real estate data has been employed to predict age indicators.

For average age, all three models are comparable, having an error smaller than two years, but LM and SVM again produce slightly better results than RF. On the other hand, SVM performs significantly poor in estimating the proportion of the elderly. Feature engineering has proven to enhance the performance and predictions for SVM (average age). Opposite of that, the results have dropped for the LM estimations.

#### Overview of the performance

LM and SVM models differ from RF by assuming linearity within the dataset [57, 69]. Throughout the study, LM has been amongst the best performing models, while SVM and RF tend to be more sensitive to the input datasets. Feature engineering is a common method in boosting ML algorithms, in particular bivariate combinations to enhance linear models. It has proven to improve some of the models. However, the estimation results with feature combinations are consistently worse within the LM model. We conclude that balancing the training data by creating a synthetic input increases the model bias, thus, implies false assumptions for the estimations [76].

Variable importance is a crucial measure in assessing ML algorithms, since it provides information about the significance of the predictors and has been widely applied in previous ML-based population studies [1, 23, 64]. Our results suggest that in general across the techniques, the number of buildings and transaction counts have the biggest effect on models in estimating population counts, while in the case of SVM, certain POIs (bus stops, committee centres and childcare facilities) tend to exhibit their importance (Table 3). LM takes all POI except clinics into account as an integral part for the estimations, whereas building counts remain the most important predictors.

**Table 3:** An overview of the predictors and their variable importance from none (o) to high (***).

Counts estimation			Age estimation				
Predictors	VarImp		Predictors	VarImp (mean age)		VarImp (senior share)	
	SVM	LM		SVM	LM	RF	LM
No. of buildings	***	***	Bldg. age (mean)	***	***	***	**
No. of transactions	***	**	Bldg. age (median)	***	*	***	**
Food establishments	*	*	Bldg. age (mode)	***	o	***	o
Supermarkets	*	*	1-Room proportion	o	*	o	*
E-waste recycling locations	o	*	2-Room proportion	o	**	*	**
Residents committees	**	*	3-Room proportion	***	***	**	***
Student care services	*	*	4-Room proportion	***	**	**	***
Childcare facilities	**	*	Executive proportion	**	o	*	o
Schools	*	*	Mean x Median	***	–	***	–
Clinics	*	o	Mode x 3-Room prop.	***	–	**	–
Bus stops	***	*					
Buildings x Transactions	***	–					
Childcare x Bus stops	***	–					

Throughout the models estimating the age patterns, building age (in particular the mean age of buildings in the subzone) and the proportion of 3- as well as 4-room flats are the most important covariates. While SVM (average age) and RF (elderly) take all building age measures into account with a high importance, the LM models tend to put more emphasis on flat types.

The scatter plots (Fig 5) reveal a nearly perfect line for population estimation and no remarkable outliers. Despite the fact that the majority of subzones contain up to 30 000 residents, also more populated areas have been predicted accurately. The dispersion of the predicted and observed age values is perceptibly bigger. For instance, one subzone (Tiong Bahru) has been overestimated by LM and SVM due to the eminently old average age of the buildings, whereas the opposite is the case for a few other subzones (e.g. Boon Keng and Depot Road). RF seems to be more robust to outliers, which can be seen in predicting the elderly proportion. But compared to LM, the estimations appear to be clustered (Fig 5). In other words, RF overestimates the lowest values, and underestimates the highest ones, which are not existing in the estimations. While SVM and LM again perform almost identically for average age predictions, there are perceptible differences between LM and RF in estimating the elderly proportion. Although the performance measures (Table 2) are similar, the distributions and individual predictions differ remarkably, particularly in terms of the lowest and highest values (Fig 5), reaffirming the importance of experimenting with multiple ML techniques.

Compared to previous studies in estimating population counts by ML methods [4, 10, 29, 46, 48], the results show high performance, especially in light of the simplicity, low computational cost, and reproducibility of our approach. The R-squared values in our method are high, meaning that a vast majority of the total variance of population numbers can be explained by real estate information (block and transaction counts) and the number of amenities. There is still a discordance in efficient measures of error (Mean Absolute Error), and therefore a wide variety of implemented measures among the different studies [72].

Given the average age of the buildings and the proportion of flat types, we were able to retrieve convincing results on predicting age structure within an administrative area. The R-squared values are still relatively high (73% for senior proportion and 75% for average age; case of RF).

Recent trends in population disaggregation diverged from linear analysis and have tackled more complex relationships between population distribution and the environment with the help of ML algorithms, especially when applying remote sensing data [1, 24, 77]. It seems evident that RF and SVM outperform simple linear models when employing numerous input datasets with various different natures and scales [4, 48]. However, it remains undecided whether it is wiser to increase the amount of highly varying predictors, rather than focusing on strong, correlated covariates, which are usually available in urban areas. In this research, across different scenarios (e.g. without and with different model tuning), LM was the best and also most constantly performing model, whereas RF and SVM have revealed a higher variance in accurately handle the training datasets. Similar trends have been found in [23], when a subset of population is included in the model.

### Limitations

There are a few limitations in our work. Most importantly, we have focused on a single city, and one with government intervention in housing, meaning that the method may not necessarily work everywhere. In our work, following the availability of data, we focus on areas that are dominated by buildings managed by the Singapore’s Housing and Development Board. Such focus is representative, as Singapore’s residential landscape is largely controlled by HDB, housing the vast majority of the nation, but it nevertheless may not give the entire picture. Perhaps including data on the remaining types of housing, which are minor but potentially demographically dissimilar, would end up with somewhat different results. Nevertheless, we believe that our pioneering work presents a contribution in investigating enhanced population estimation.

This study was also limited to the available predictors and the spatial resolution of the census data for validation. For example, eldercare facilities, which might have been important, could not be included in the research due to their low number in the study area. Although our method can be applied on individually adjustable areas, POI with smaller numbers (such as schools or committee centres) will become less important for higher resolutions, while we expect age distributions based on real estate data to remain meaningful for smaller areas. One of the likely causes why amenities have not been useful in predicting age is that they are used by residents from nearby districts as well, and they do not exclusively cater to the subset of the population living in public housing.

## Conclusion

Our study highlights the ability of different ML techniques to estimate population counts, average age, and elderly proportion by spatially detailed knowledge on POI and real estate data. Our three main takeaways and contributions are:

- Traditional population estimation techniques may be enhanced to reveal demographic properties of neighbourhoods beyond just the number of residents, which has been the main focus of related work. Our work demonstrates that age distribution can be predicted with high accuracy.
- Real estate data beyond the conventionally used housing stock, such as the amount of property transactions and flat types, adds value to the estimations. We encourage researchers in related work to make use of such data when available, as some of the impactful predictors featured in this paper have not been used previously in the realm of population estimation.
- Variables extracted from amenities, as the traditionally used predictors for population estimation (the importance of which we affirm in our population count predictions), appear not to be useful for estimating age, at least in our proof of concept developed at the fine spatial scale in the case of Singapore. The successful estimations of age have rather been achieved thanks to real estate data, e.g. flat type distribution and age of buildings.

The methods of this work could be applied on similar urban areas to support city planners and decision makers facing future challenges. Real estate data has proven to be a strong indicator for demographic patterns, and it would be interesting to analyse if similar correlations could be found in other cities. Due to the simplicity and implementation of the techniques that have been used, the predictors could be altered, extended or combined with little effort. The results allow to take efficient action in questions, which are directly linked to population density and age patterns, such as transportation, infrastructure, and education.

For future work, it would be beneficial to research whether other demographic characteristics such as income, education level, gender ratio, and ethnicity could be predicted as well. Even though in our work amenities have not been useful in predicting age, perhaps they might be reliable predictors of other demographic characteristics and they would be more useful in other locations. Further, we plan to add the temporal dimension in our experiments, e.g. investigate whether the developed approach can estimate the age change over time.

It would also be interesting to investigate whether other forms of real estate and urban data could contribute to such estimations, and whether demographics could be sensed already from property ads (rent and sale), before actual property transactions occur, as a predictor of population dynamics and changes in the foreseeable future. Further examples of urban data that may be investigated pertain to vibrancy, which are increasingly used in other domains of urban analytics [78, 79].
